# Cost outcome analysis of decentralized care for drug-resistant tuberculosis in Johannesburg, South Africa

**DOI:** 10.1371/journal.pone.0217820

**Published:** 2019-06-06

**Authors:** Craig van Rensburg, Rebecca Berhanu, Kamban Hirasen, Denise Evans, Sydney Rosen, Lawrence Long

**Affiliations:** 1 Department of Internal Medicine, School of Clinical Medicine, Faculty of Health Sciences, University of the Witwatersrand, Johannesburg, South Africa; 2 Health Economics and Epidemiology Research Office, Wits Health Consortium, University of Witwatersrand, Johannesburg, South Africa; 3 Department of Global Health, School of Public Health, Boston University, Boston, United States of America; Médecins Sans Frontières (MSF), SOUTH AFRICA

## Abstract

**Background:**

Drug resistant-tuberculosis is a growing burden on the South African health care budget. In response the National Department of Health implemented two important strategies in 2011; universal access to drug-sensitivity testing for rifampicin with Xpert MTB/RIF as the first-line diagnostic test for TB; and decentralization of treatment for RR/MDR-TB to improve access and reduce costs of treatment.

**Objective:**

Estimate the costs by treatment outcome of decentralized care for rifampicin and multi-drug resistant tuberculosis under routine conditions. The study was set at an outpatient drug resistant-tuberculosis treatment facility at a public academic hospital in Johannesburg, South Africa. During the study period 18–24 month long course treatment was offered for rifampicin-resistant and multi-drug-resistant tuberculosis.

**Methods:**

Data are from a prospective observational cohort study. Costs of treatment were estimated from the provider perspective using bottom-up micro-costing. Costs were estimated as patient-level resource use multiplied by the unit cost of the resource. Clinic visits, drugs, laboratory tests, and total days hospitalized were collected from patients’ medical records. Staff time was estimated through a time and motion study. A successful treatment outcome was defined as cure or completion of the regimen.

**Results:**

We enrolled 124 patients with 52% having a successful outcome. The average total cost/patient for all patients was $3,430 and $4,530 for successfully treated patients. The largest contributors to total cost across all outcomes were drugs (43%) and staff (28%). The average cost to achieve a successful outcome including all patients who started treatment (“production cost”) in the cohort is $6,684.

**Conclusions:**

Decentralized, outpatient RR/MDR-TB care under South Africa’s 2011 strategy costs 74% less per patient than the previous strategy of inpatient care. The treatment cost of RR/MDR-TB is primarily driven by drug and staff costs, which are in turn dependant on treatment length.

## Introduction

Drug-resistant tuberculosis (DR-TB) threatens achievement of global TB control. Despite the decline in new TB cases globally, rates of rifampicin (RIF) resistant and multi-drug resistant tuberculosis (RR/MDR-TB) are increasing [1]. Access to treatment is limited and treatment outcomes remain extremely poor; the World Health Organization (WHO) estimates that only 12% of an estimated 600,0000 annual cases of RR/MDR-TB globally are successfully treated [1]. Along with poorer outcomes, treatment costs of RR/MDR-TB are much higher than those of drug susceptible TB, with the main driver being the cost of inpatient care [1,2].

The rates of RR/MDR-TB have been increasing in South Africa from 3.4% in the first national drug drug-resistance survey undertaken in 2001–2002 to 4.6% in the most recent 2012–2014 survey [3]. This increase in drug-resistant TB represented a huge burden on the health system, as the cost of hospital-based RR/MDR-TB care was nearly 40 times the cost of treating drug-susceptible TB [4]. In response, the South African National Department of Health implemented two important strategies in 2011: universal access to drug-sensitivity testing for rifampicin with Xpert MTB/RIF as the first-line diagnostic test for TB; and decentralization of treatment for RR/MDR-TB to improve access and reduce costs of treatment [5,6]. These policies have resulted in a dramatic increase in the number of patients diagnosed with RR/MDR-TB, from 8,072 in 2011 to 19,073 in 2016 [1,7]. While this increase represents a success in case-finding, it also places a growing burden on the budget of the national TB programme. Diagnosis and treatment of RR/MDR-TB were estimated to account for a third of the total TB programme budget even before 2011 [8]; if costs increase in proportion to cases, far more funding will be needed. Determining the cost implications of the new guidelines is important if South Africa is to effectively implement its TB programme.

Based solely on the guidelines, successful treatment of a case of MDR-TB has been estimated to cost $3,141 (adjusted to an exchange rate of ZAR13.31/USD) [9]. This estimate assumed, however, that all patients were treated precisely according to guidelines, with no variation among patients based on condition, logistical challenges, or other potential variants. Early estimates of the cost to successfully treat RR/MDR-TB through a decentralized, community-based programme were substantially higher than estimates based on guidelines only: $5,531 a 2015 study in Khalyelistsha, near Cape Town [8] and $5,286 from a 2018 study based in KwaZulu Natal [10]. These studies reflect conditions very soon after the guideline changes, though, and may no longer represent true costs incurred. In this study we add to this literature by describing the cost and outcomes of decentralized care for RR/MDR-TB under routine conditions based on recent experience managing DR-TB at a public sector treatment site in Johannesburg, South Africa.

## Methods

### Study site and population

The study took place at an outpatient DR-TB treatment facility at a public academic hospital, in Johannesburg, South Africa. We included adult patients (>18 years) from a larger prospective observational cohort study of RR-TB patients at the same facility (Wits HREC protocol M130205), who initiated RR/MDR-TB treatment between March 2013 and September 2016 [11]. Patients were diagnosed and referred from an inpatient ward or one of the primary health clinics in the hospital’s catchment area. To describe costs and outcomes of decentralized care, patients referred from the ward were included in the study if they had received less than a month of inpatient care for TB. Patients diagnosed with extensively drug-resistant TB (pre-XDR/XDR-TB) were excluded from the analysis, as were patients who were transferred to another facility prior to reaching a study endpoint.

### RR/MDR-TB treatment programmes

Care at the study facility followed the 2013 South African National DR-TB guidelines [6,12]. During the period of this analysis patients were treated with long course therapy, consisting of six months of injectable therapy with kanamycin and 18–24 months of oral therapy with moxifloxacin, terizidone, pyrazinamide, and either ethionamide or isoniazid depending on the isoniazid resistance mutation. Patients with *katG* mutation received ethionamide and those with the *inhA* mutation were treated with high dose isoniazid. Patients with rifampicin mono-resistant TB (RMR) also had isoniazid substituted for ethionamide. Patients who experienced toxicity to the standard regimen were treated with individualised regimens that usually included para-aminosalicylic acid (PAS). All patients’ care was primarily managed through the outpatient facility, but kanamycin was administered at primary health care clinics near to the patient’s home three or five days per week, depending on the prescribed dosage. This reduced the number of times patients were required to travel to the outpatient treatment facility. New and repurposed drugs such as bedaquiline, linezolid and clofazimine only became available in 2015 so were not in use during the study period [13]. Patients diagnosed with additional resistance, either pre-XDR or XDR were transferred to the provincial centralised DR-TB hospital for individualised inpatient treatment. Antiretroviral therapy (ART) was fully integrated into DR-TB care at the treatment site.

Patients with isoniazid and rifampicin resistance detected on either genotypic (Xpert MTB/RIF or line probe assay) or phenotypic drug susceptibility testing were categorized as multi-drug resistant (MDR), and those with isolated rifampicin resistance are rifampicin mono-resistant (RMR). Rifampicin resistance by Xpert (RR-TB by Xpert) is a term used to describe patients without isoniazid susceptibility testing due to baseline negative or contaminated cultures. During the study period line probe assays were not routinely done on raw specimen or on contaminated specimens so these patients had no further susceptibility testing results.

TB treatment outcomes, contained in Box 1, were defined according to the 2009 South African National Tuberculosis Control Programme guidelines and the 2013 WHO Definitions and Reporting Framework for Tuberculosis as cured, treatment completed, treatment failed, lost to follow-up (LTFU), died, transferred-out or still in treatment. Standard WHO treatment outcomes are measured at 24 months, and that is the also standard in the published literature. However, in this analysis treatment outcomes were evaluated at two time points: 24 months and 36 months due to significant number of patients still on treatment at 24 months who went on to complete therapy by 36 months. Patients who were cured or completed treatment were deemed to have had a successful final outcome.

**Box 1. Treatment outcome definitions**

TB treatment outcomes are mutually exclusive and for the purpose of this analysis the final outcome was defined at 36 months except in the case of loss to follow up, treatment failure and death, which were defined when they occurred. TB treatment outcomes were defined according to the 2009 South African NTP guidelines and the 2013 WHO Definitions and Reporting Framework for Tuberculosis as: 1) Cured. Patients considered cured have no evidence of failure AND three or more consecutive cultures taken at least 30 days apart are negative after the intensive phase. 2) Treatment completed. Treatment completion refers to patients who complete treatment but do not meet the criteria to be classified as cured or as treatment failure. Treatment success is the sum of cured and treatment completed. 3) Treatment failed. Treatment failure refers to patients whose treatment is terminated or a permanent regimen change of at least two anti-TB is required because (i) lack of conversion by the end of the intensive phase (max 8 months), (ii) bacteriological reversion in the continuation phase after conversion to negative, (iii) drug sensitivity tests indicate additional acquired resistance to fluoroquinolones or second-line injectable drugs and (iv) adverse drug reactions. 4) Lost to follow-up. Those who miss more than two consecutive months of treatment are considered to be lost to follow-up (LTF). 5) Died. Death includes all-cause mortality during the course of treatment. 6)Transferred out. Outcomes will not be evaluated for patients with a record of transferring to another facility and no treatment outcome assigned.

### Cost analysis

Costs of treatment of RR/MDR-TB were estimated from the provider perspective, using bottom-up micro-costing and included drugs, laboratory tests, staffing, equipment, consumables, overheads (electricity, water and effluent), and inpatient days. We estimated patient resource use from the day of treatment initiation until a final outcome was obtained. We distinguished two different types of visits which occurred during the intensive phase of treatment; those to the outpatient treatment facility, and those to the local primary health care clinic where daily injectable kanamycin was administered. Costs were estimated as resource use (total units) multiplied by the unit cost of the resource. The cost of diagnosis of RR/MDR-TB and the cost of (ART) were not included in our analysis.

All unit costs were in 2017 South African Rand (ZAR) or adjusted to 2017 costs. The results were converted to United States Dollars (USD) at the average 2017 exchange rate of 13.31 ZAR to 1 USD.

## Resource usage and costs

From patient files we collected the total number and types visits to the main treatment clinic, TB and non-TB drugs dispensed, laboratory tests performed, audiology visits and total inpatient days reported. As patient records do not contain sufficient detail to estimate a unique visit cost based on actual resources used we estimated the average resource use for these resources per visit type (e.g. outpatient treatment facility or local primary health care clinic).

Staff time for visits in each phase of treatment was estimated using time and motion data collected with staff completed forms, detailing the start and end time of the visit. The following interactions with staff at the main treatment site were measured: measuring of weight and vital signs, consultation with a doctor, time spent with a nurse, counselling by a lay counsellor, time taken to administer a kanamycin injection, hearing test and time with audiologist, medication dispensing by a pharmacist. In addition, patients also routinely had blood work done, and had a baseline chest X-ray at the start of treatment. The staff cost per minute was calculated using public sector salaries reported for health care workers at the respective levels. We estimated the consumables, such as needles and gloves, used per visit type through discussion with the staff and mapping what activities occur during a visit. Unit costs of consumables were obtained from supplier price lists and public tender documents. Equipment costs were obtained from clinic records or written quotes and were annualised at a discount rate of 3%. Total overhead costs were estimated by multiplying the size of the facility by an estimated cost per square meter. Cost per square meter were estimated from records obtained from a private company in the same area.

We estimated the number of visits patients make to their local primary health care clinic based on the prescribed dose of kanamycin. We estimated the cost of the staff time and materials required to administer kanamycin injections using the time and motion data from the outpatient treatment facility. This information was then used as a proxy for the cost of injection administration at the patients’ local primary health care clinics.

We assumed consumables use was the same as visits in the outpatient treatment facility, and included time taken for a nurse to administer the injection. Equipment and overhead costs for the local primary health care clinic visit were added as a mark-up of 10% over the staff and consumables costs for the visit.

Drug costs were taken from the National Department of Health’s master procurement catalogue. Costs were calculated by the drugs prescribed, the dosage required and the duration the drug would be taken for. Patients who were stable on treatment and demonstrated good adherence were provided two months of drugs at each visit in the continuation phase. Laboratory and monitoring costs were obtained from the National Health Laboratory Service’s state price list for 2017. Inpatient days included hospitalization at the time of diagnosis, and any other hospital admissions that occurred during treatment due to adverse events, worsening clinical condition or other illnesses. Hotel costs for inpatient days were provided by the Health System’s Trust District Health Barometer for 2016/2017.

## Ethics

Ethical approval was granted by the Human Research Ethics Committee at the University of Witwatersrand. Participants provided written informed consent to participate in this study.

## Results

Baseline demographics and clinical characteristics are presented in Table 1. A total of 124 patients were included in the final cohort. Half were female 62/124 (50%) with a median age 38 (IQR 31–42.5). The majority of patients were HIV co-infected 109/124 (87.8%) with a median CD4 count 107 cells/mm^3^ (IQR 27–274) at treatment initiation. Half of HIV-positive patients were on ART at time of RR/MDR-TB treatment initiation (n = 54/109; 50.5%), for a median of 11.4 months (IQR 3.8–29.9). Nearly half of the patients in the cohort had rifampicin mono-resistant TB (RMR) 60/124 (48.3%), 38/124 (30.6%) had RR/MDR-TB, and 26/124 (21%) had rifampicin resistant TB diagnosed by Xpert without further confirmatory testing (RR-TB by Xpert). A larger proportion of MDR-TB patients (79%) were referred from outpatient facilities than RMR (57%) and RR-TB by Xpert (54%). RR-TB by Xpert also had the lowest proportion of smear positive (3.8%) vs 17% and 26% for RMR and MDR-TB respectively. RR-TB by Xpert patients were managed as though they were confirmed MDR-TB patients.

**Table 1:** Baseline demographic and clinical characteristics of DR-TB patients included in 36 month outcomes analysis (n = 124).

		RR-TB by Xpert	Rif-Mono	MDR	Total
Variable	Description	N = 26	N = 60	N = 38	N = 124
Sex	Male	13/26 (50.0%)	35/60 (58.3%)	14/38 (36.8%)	62/124 (50.0%)
	Female	13/26 (50.0%)	25/60 (41.7%)	24/38 (63.2%)	62/124 (50.0%)
Age at Initiation (years)	Median (IQR)	37.0 (33.0–41.0)	38.0 (30.5–42.0)	37.5 (29.0–46.0)	38.0 (31.0–42.5)
	18–29	4/26 (15.4%)	14/60 (23.3%)	10/38 (26.3%)	28/124 (22.6%)
	30–49	21/26 (80.8%)	38/60 (63.3%)	24/38 (63.2%)	83/124 (66.9%)
	≥50	1/26 (3.9%)	8/60 (13.3%)	4/38 (10.5%)	13/124 (10.5%)
Employment	Employed	13/26 (50.0%)	32/59 (54.2%)	14/38 (36.8%)	59/123 (48.0%)
	Unemployed	13/26 (50.0%)	27/59 (45.8%)	24/38 (63.2%)	64/123 (52.0%)
HIV Status	Negative	2/26 (7.7%)	9/60 (15.0%)	4/38 (10.5%)	15/124 (12.1%)
	Positive: On ART Positive: Not on ART Positive: Unknown	13/26 (50.0%) 9/26 (34.6%) 2/26 (7.7%)	27/60 (45.0%) 22/60 (36.7%) 2/60 (3.3%)	14/38 (36.8%) 20/38 (52.6%) 0/38 (0.0%)	54/124 (43.5%) 51/124 (41.1%) 4/124 (3.2%)
CD4 Cell Count (cells/mm³)	Median (IQR)	86.5 (30.5–193.5)	87.5 (24.5–188.5)	139.0 (41.0–437.0)	107.0 (27.0–274.0)
	≤50	7/24 (29.1%)	16/51 (31.3%)	10/35 (28.6%)	33/109 (30.2%)
	51–100	6/24 (25.0%)	10/51 (19.6%)	2/35 (5.7%)	18/109 (16.5%)
	>100	11/24 (45.8%)	22/51 (43.1%)	23/35 (65.7%)	56/109 (51.3%)
	Unknown	2/24 (8.3%)	2/51 (3.9%)	0/35 (0%)	4/109 (3.7%)
Time on ART (months)	Median (IQR)	10.2 (2.2–29.0)	11.4 (5.0–29.9)	16.0 (4.3–40.1)	11.4 (4.0–29.9)
	≥6	7/13 (53.8%)	19/27 (70.4%)	9/13 (69.2%)	35/53 (66.0%)
	<6	6/13 (46.2%)	8/27 (29.6%)	4/13 (30.8%)	18/53 (34.0%)
Referring Facility	Outpatient	14/26 (53.9%)	34/60 (56.7%)	30/38 (79.0%)	78/124 (62.9%)
	Inpatient	12/26 (46.2%)	26/60 (43.3%)	8/38 (21.1%)	46/124 (37.1%)
Patient Category	New	16/26 (61.5%)	29/60 (48.3%)	31/38 (81.6%)	76/124 (61.3%)
	Previously treated, 1^st^ line drugs	8/26 (30.8%)	25/60 (41.7%)	5/38 (13.2%)	38/124 (30.7%)
	Previously treated, 2^nd^ line drugs	2/26 (7.7%)	6/60 (10.0%)	2/38 (5.3%)	10/124 (8.1%)
TB Type	PTB + EPTB	4/26 (15.4%)	10/60 (16.7%)	4/38 (10.5%)	18/124 (14.5%)
	EPTB only	4/26 (15.4%)	8/60 (13.3%)	4/38 (10.5%)	16/124 (12.9%)
	PTB only	18/26 (69.2%)	42/60 (70.0%)	30/38 (79.0%)	90/124 (72.6%)
Smear Microscopy	Negative	24/26 (92.3%)	46/60 (76.6%)	24/38 (63.2%)	94/124 (75.8%)
	Positive	1/26 (3.8%)	10/60 (16.7%)	10/38 (26.3%)	21/124 (16.9%)
	Missing	1/26 (3.8%)	4/60 (6.7%)	4/38 (10.5%)	9/124 (7.2%)

We present patient outcomes at 24 and 36 months follow up Table 2. By 24 months 23/124 patients (19%) were still in treatment, of whom 20 went on to have a successful outcome by 36 months. A total 64/124 (51.6%) patients had a successful outcome over the 36-month follow up period, with outcomes only differing marginally between the resistance types. However, 29% of patients with MDR-TB were still on treatment at 24 months, against 17% of RMR and 12% of RR-TB by Xpert. Patients diagnosed as RR-TB by Xpert had a lower rate of death than those with RMR and MDR-TB. Baseline characteristic of the patients, such as HIV status or gender, did not have an effect on the outcomes.

**Table 2:** Patient outcomes by resistance type.

	RMR N = 60	RR-TB by Xpert N = 26	MDR N = 38	Total N = 124	RMR N = 60	RR-TB by Xpert N = 26	MDR N = 38	Total N = 124
	At 24 months				At 36 months			
Still in treatment	10 (17%)	3 (12%)	10 (29%)	23 (19%)	0 (0%)	0 (0%)	0 (0%)	0 (0%)
LTFU	15 (27%)	9 (35%)	10 (26%)	34 (27%)	17 (27%)	10 (38%)	10 (26%)	37 (30%)
Died	13 (22%)	2 (8%)	8 (21%)	23 (19%)	13 (22%)	2 (8%)	8 (21%)	23 (19%)
Completed	13 (22%)	4 (15%)	6 (16%)	23 (19%)	18 (30%)	5 (19%)	11 (29%)	34 (27%)
Cured	9 (15%)	8 (30%)	4 (11%)	21 (17%)	12 (21%)	9 (35%)	9 (24%)	30 (24%)
Success*	*22 (37%)*	*12 (45%)*	*10 (24%)*	*44 (35%)*	*30 (51%)*	*14 (54%)*	*20 (53%)*	*64 (52%)*

*Treatment success = the sum of cured and treatment completed.

Table 3 provides the cost breakdown by the different resistance patterns, for each of the outcomes. Drug costs are the largest cost driver, making up between 40 and 50% of the total cost for successfully treated patients, followed by staff costs which make up between 25 and 30%. MDR-TB patients are more expensive to treat, costing $150 more to complete treatment and $785 more per cure than RMR. Patients diagnosed RR-TB by Xpert cost the least to obtain a successful outcome ($4,178).

**Table 3:** Cost breakdown by outcome and TB resistance pattern (2017 USD).

Outcome	Resistance pattern	Staff cost	Drug cost	Labs and monitoring	Equipment	Consumables	Overheads	Inpatient	Average (SD)	Median (IQR)
All patients	RMR	917	1,205	332	85	25	70	858	3,492 (2,383)	3,257 (1,914–4,287)
	RR by Xpert	870	1,169	281	86	27	70	737	3,239 (1,796)	3,210 (1,774–4,081)
	MDR	892	1,527	327	85	25	68	572	3,495 (2,622)	3,314 (1,057–5,322)
Treatment success	RMR	1,295	1,785	388	124	30	98	732	4,450 (1,984)	3,845 (3,392–5,645)
	RR by Xpert	1,114	1,640	362	113	33	91	827	4,178 (1,352)	3,631 (3,217–4,641)
	MDR	1,332	2,375	435	122	34	101	499	4,898 (1,881)	4,228 (3,389–6,395)
Completed treatment	RMR	1,315	1,681	378	126	29	98	732	4,359 (1,411)	3,961 (3,445–4,951)
	RR by Xpert	1,046	1,471	376	115	35	96	1,038	4,176 (1,197)	4,019 (3,353–4,019)
	MDR	1,262	2,277	383	116	32	96	345	4,509 (2,191)	3,989 (2,957–5,322)
Cured	RMR	1,264	1,941	402	120	32	96	732	4,587 (1,838)	3,835 (3,078–6,170)
	RR by Xpert	1,151	1,734	354	112	31	88	710	4,179 (1,711)	3,567 (3,195–4,383)
	MDR	1,417	2,495	499	130	36	108	687	5,372 (1,564)	5,731 (4,184–6,456)
Defaulted	RMR	620	733	270	53	22	49	352	2,099 (1,625)	2,011 (726–2,723)
	RR by Xpert	672	717	199	61	22	52	659	2,381 (1,533)	1,964 (1,428–3,010)
	MDR	555	890	240	55	19	43	619	2,421 (2,802)	1,609 (457–3,353)
Died	RMR	432	482	285	39	19	35	1,811	3,104 (3,638)	1,624 (826–3,112)
	RR by Xpert	152	136	122	28	9	13	499	958 (529)	957 (584–1332)
	MDR	212	204	163	29	8	16	698	1,331 (1,859)	461 (355–1,509)

The cost difference between MDR, RMR and RR-TB by Xpert is related to average treatment length. Successfully treated patients with MDR were in treatment for an average of 709 days, vs 660 for RMR and 607 for RR-TB by Xpert (Table 4). The duration of treatment was related to the time to culture conversion which was on average 103 days for MDR, 61 days for RMR and 24 days for RR-TB by Xpert. We found that the average cost, across the three groups, to successfully treat a patient in an outpatient, decentralized DR-TB clinic was $4,530.

**Table 4:** Treatment length by resistance pattern (days).

Outcome	Resistance pattern	Average treatment length, days (SD)
All patients	RMR	452 (279)
	RR by Xpert	457 (264)
	MDR	437 (333)
Treatment success	RMR	660 (103)
	RR by Xpert	607 (118)
	MDR	709 (105)
Completed treatment	RMR	665 (104)
	RR by Xpert	645 (146)
	MDR	702 (110)
Cured	RMR	632 (104)
	RR by Xpert	586 (103)
	MDR	717 (103)
Defaulted	RMR	324 (257)
	RR by Xpert	337 (239)
	MDR	217 (254)
Died	RMR	162 (222)
	RR by Xpert	10 (14)
	MDR	32 (74)

## Discussion

We performed a bottom-up micro-costing analysis of treatment of RR/MDR-TB in an outpatient, decentralized model of care in South Africa. The proportion of successfully treated patients in the three groups were not significantly different, with only a 3% range between them. The average cost to successfully treat a patient was $4,530, a 74% reduction in costs compared to the inpatient model, which was shown to cost over $17,000 [4]. As the average success rate was 52%, total cost per treatment success including all patients who started treatment (“production cost”) is $6,684. The difference between these two figures represents costs for patients with unsuccessful outcomes, suggesting that overall treatment program cost will increase by more than the cost of treatment to achieve a higher number of successful treatments, unless a more effective treatment program is implemented.

Patient outcomes for decentralized care in our results are similar to those of previous studies on decentralized care, and are also similar to outcomes achieved from hospital based care [8,10]. Our estimates for the cost of successful treatment for MDR-TB through the decentralized programme are, on average, lower than other estimates based on patient data, but higher than the estimate based on treatment guidelines only [8–10]. Costs of RR/MDR-TB treatment were variable in our study, both across resistance profiles as well as within them. The largest difference in treatment costs was between patients with MDR-TB patients and those with RR-TB by Xpert, particularly for those cured where the difference was $1,200.

The treatment cost of RR/MDR-TB is primarily driven by drug and staff costs, which accounted for 43% and 28% of the total cost respectively. These costs vary with the duration of treatment required—longer periods of care require more visits and medications. RR-TB by Xpert cases were less costly to treat than MDR-TB cases, despite receiving the same regimen, as they had the shortest overall treatment time. This is likely a group of patients with lower bacterial burden at baseline which is why confirmatory drug sensitivity testing could not be obtained prior to culture conversion.

While not one of the objectives of this study we note a large proportion of the patients were HIV-positive and receiving ARVs. Average monthly ARV costs did not vary significantly between the groups, averaging $9,40 with most patients receiving a regimen of tenofovir, lamivudine and efavirenz We chose to report the RR/MDR-TB costs separately as the cost of ART provision is well documented in South Africa. The conditional grant in South Africa also has money budgeted separately for HIV and TB so it is useful to have these costs separated for national budget modelling and planning purposes. Previous literature is also mixed with regards to reporting ARV costs with RR/MDR-TB treatment costs in South Africa [4,8,9,14,15].

Since the introduction of decentralized care, South Africa has stated its intention to move to a shortened regimen for the treatment of RR/MDR-TB, as well as the introduction of bedaquiline as a substitute for kanamycin [13,16]. As we found drug costs to be the biggest contributor to the total costs, the switch to bedaquiline will have significant cost implications, which may be offset by the shortened regimen duration.

This analysis is subject to a number of limitations. This is a single site study which only included adults, serving an urban population so it may not be relevant to all settings. We relied on routine clinic data and some resource use may not have been recorded. We were also costing a single disease in a clinic which provided integrated care, and we were unable to distinguish staff time spent for TB alone from staff time on integrated care for the HIV-positive patients. This may mean our staff cost estimates are higher than would be expected in a low HIV prevalence setting or where care is not integrated. Patient costs were also not included. Outpatient care shifts more costs onto patients, who incur expenses for multiple outpatient clinic visits. However, it also allows them to remain active parts of their family and community and potentially return to employment, which may mitigate the additional travel costs. The economic effects of a decentralized model will thus depend on the community it serves. There were also too few HIV-negative patients for us to significantly compare outcomes and costs to the HIV-positive patients.

Despite these limitations, this study provides a robust estimate of the current cost of decentralized RR/MDR-TB treatment and, consequentially, potential opportunities to reduce this cost. This information will support budgeting and financial planning for DR-TB services as South Africa scales up DR-TB case-finding and access to services to achieve national and international targets.
